# Effects of COVID-19 Emergency Alert Text Messages on Practicing Preventive Behaviors: Cross-sectional Web-Based Survey in South Korea

**DOI:** 10.2196/24165

**Published:** 2021-02-25

**Authors:** Minjung Lee, Myoungsoon You

**Affiliations:** 1 Department of Public Health Sciences, Graduate School of Public Health Seoul National University Seoul Republic of Korea; 2 Office of Dental Education, School of Dentistry Seoul National University Seoul Republic of Korea

**Keywords:** COVID-19, coronavirus, preventive behaviors, text message, mobile phone, alert, prevention, behavior, public health, survey

## Abstract

**Background:**

Sending emergency messages via mobile phone text messaging can be a promising communication tool to rapidly disseminate information and promote preventive behavior among the public during epidemic outbreaks. The battle to overcome COVID-19 is not yet over; thus, it is essential that the public practices preventive measures to prevent the spread of COVID-19.

**Objective:**

This study aimed to investigate the effectiveness of reading and obtaining information via emergency alert SMS text messages and their effects on the individual's practice of preventive behaviors during the early stages of the COVID-19 outbreak in South Korea.

**Methods:**

A cross-sectional web-based survey comprising 990 participants was conducted over 3 days (March 25-27, 2020). A multivariable logistic regression analysis revealed the sociodemographic factors that might influence the behavior of reading emergency alert text messages. A hierarchical linear regression model estimated the associations between reading emergency alert text messages for each precautionary behavior practiced against COVID-19. Additionally, the indirect effects of reading the text messages on each precautionary behavior via psychological factors (ie, perceived risk and response efficacy) were calculated. All data were weighted according to the 2019 Korea census data.

**Results:**

Overall, 49.2% (487/990) of the participants reported that they always read emergency alert text messages and visited the linked website to obtain more information. Factors such as female sex (odds ratio [OR] 1.68, 95% CI 1.28-2.21) and older age (30-39 years: OR 2.02, 95% CI 1.25-3.28; 40-49 years: OR 2.84, 95% CI 1.80-4.47; 50-59 years: OR 3.19, 95% CI 2.01-5.06; 60 years and above: OR 3.12, 95% CI 2.00-4.86 versus 18-29 years) were identified to be associated with a higher frequency of reading the text messages. Participants who always read the text messages practiced wearing facial masks (β=.074, *P=*.01) more frequently than those who did not. In terms of social distancing, participants who reported they always read the text messages avoided crowded places (β=.078, *P=*.01) and canceled or postponed social gatherings (β=.103, *P<*.001) more frequently than those who did not read the text messages. Furthermore, reading text messages directly and indirectly affected practicing precautionary behaviors, as the mediation effect of response efficacy between reading text messages and practicing preventive behaviors was significant.

**Conclusions:**

Our findings suggest that emergency alert text messages sent to individuals' mobile phones are timely and effective strategies for encouraging preventive behavior in public. Sending emergency alert text messages to provide the public with accurate and reliable information could be positively considered by the health authorities, which might reduce the negative impact of infodemics.

## Introduction

In recent years, mobile technology and text messages have emerged as a promising communication tool to rapidly disseminate information during several emergencies [1-3]. There is a widespread use of and access to smartphone and mobile devices, with mobile phone technology penetration at nearly 100% worldwide. South Korea has among the highest ownership percentages (94%) [4]. Their improved geolocation capacity and access to broadband and satellite communication infrastructure have enabled emergency alert SMS text messages to be sent to end users directly [5]. One of the attractive features of mobile text communication for emergency communication is the ability to target text messages to all phones in a specific location very quickly [6,7], making such communication highly efficient. Moreover, unlike some other media sources, readers can read, reread, and analyze the information provided via text messages [8]. A previous study in the United Kingdom suggests that a system that sends emergency messages via mobile phone text messaging would be generally well accepted by the public and likely to improve uptake of protective behaviors when combined with other approaches [9].

After the outbreak of Middle East Respiratory Syndrome (MERS) in 2015, South Korea passed the Framework Act on the Management of Disasters and Safety. This Act established disaster and safety management systems for state and local governments and prescribed matters necessary for disaster management to protect citizens' lives, bodies, and properties. The Act included new regulations for emergency management and response to improve safety, such as requiring owners or managers of telecommunication facilities’ preferential capacity to forecast, alert, notify, or undertake other emergency measures concerning a disaster when necessary. The central and local governments of South Korea have sent emergency alert text messages to the public during the COVID-19 outbreak. In general, emergency alert text messages include two types of content. In the first type, the central government sends persuasive messages, encouraging individuals to take preventive measures. In the second type, local governments also recommend precautionary behaviors but mostly send risk information such as the number of confirmed cases in the residence area, contact tracing of confirmed cases, and closure of certain places. Examples of text messages sent to the public in March 2020 are shown in Table 1.

**Table 1:** Examples of emergency alert text messages sent to the public during the COVID-19 pandemic.

Sender and message contents (translated)		Date sent
**Central governments (Central Disaster and Safety Countermeasures Headquarters of Korea)**		
	*Join us for a“two-week stop”to protect your family and friends! Thank you for your dedication.* [10]	March 23, 2020
	*Observe strict hygiene rules when caring for the elderly.* *Even if you are apart, your heart is close.* [11]	March 24, 2020
**Local governments**		
	*Three additional COVID-19 confirmedcases occurred. An epidemiological investigation is currently underway, and the route of confirmed persons is scheduled to be released on the website after confirmation.* (Seocho City Office)	March 13, 2020
	*Check the route of the 5th COVID-19 confirmed case of Jeollanam-do (Hwasun) on the county office's website, and if the route overlaps, please consult with Jangseong Community Health Center (061-360-8333).*	March 17, 2020

The battle to overcome COVID-19 is not yet over. Therefore, nonpharmaceutical interventions such as wearing facial masks and practicing social distancing are critical. Efforts to sustain and elevate these practices by the public are among the most important goals of public health authorities. Effective public health risk communication about the outbreak and guidance on how to respond can alleviate the negative impacts of the public health emergency and save lives [12]. There can be negative consequences if the public does not practice preventive behaviors quickly enough [13]. Thus, public health education and public health policies should be implemented so that “social learning,” which may be described as the collective effects of communication efforts, can take place since the public is one of the most critical stakeholders in combatting the outbreak [13,14].

Successful emergency communication is determined by how quickly or reliably a message can be disseminated and how people respond to the information they receive [15,16]. Emergency alert messages have no benefit unless the readers read the message and follow the provided guidelines. Therefore, understanding why people accept or resist responding to warning and alert messages is essential. Some researchers have proposed decision models that could harmoniously explain recipient behaviors in response to emergency alerts [17-20]. These models include protective action decision-making [18], protection motivation theory [20], person-relative-to-event theory [21], and the theory of planned behavior [17]. According to the protection motivation theory, threat appraisal depends on individual perceptions of disease severity (ie, evaluating the state of the environment and observing what happens to others) and the individual’s susceptibility. A coping appraisal is driven by the efficacy belief, which comprises perceived response efficacy (ie, the belief that the recommended behavior will protect) and self-efficacy (ie, the ability to perform the recommended behavior). We examined two psychological concepts by adopting the protection motivation theory to explore preventive behaviors among individuals—perceived risk and response efficacy.

**Figure 1:**
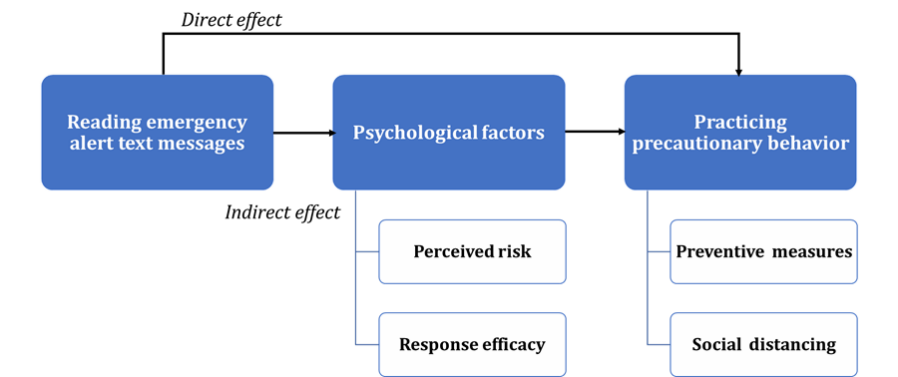
Framework used in this study.

In this study, we investigated the effectiveness of reading and obtaining information from emergency alert text messages and their effects on the individual's preventive behaviors during the COVID-19 outbreak in South Korea. First, we examined the extent to which people read the text messages and identified sociodemographic factors that contribute to this behavior. Second, we determined whether the individuals responded to these messages by investigating the direct effect of reading text messages on practicing preventive behaviors. Finally, we examined whether the effect of reading text messages on practicing precautionary behaviors was mediated via psychological factors by investigating indirect effects through perceived risk of COVID-19 infection and response efficacy of each behavior. The framework of this study was constructed as shown in Figure 1. To our knowledge, most previous studies evaluating the effect of emergency alert text messages depended on behavioral intentions, mainly in hypothetical emergencies [9,22-24]. These previous studies are limited because behavioral intent in hypothetical situations may not accurately predict behavioral responses in actual situations [25,26].

## Methods

### Study Design and Sampling

A cross-sectional web-based survey design was adopted to (1) evaluate preventive behaviors of the public and (2) assess whether they read emergency alert text messages during the COVID-19 pandemic, by using an anonymous questionnaire. The survey was conducted via a web-based platform developed by a research company called Korea Research. The company recruited respondents by sending survey invitations containing general information about the survey, such as its aim and consent statement via email or text messages, to registered survey panel members who met the inclusion criteria. The inclusion criteria were as follows: (1) aged 18 years or older, (2) a resident of South Korea, and (3) a Korean speaker.

The recruiting company sampled respondents using a proportional quota sampling process based on age, sex, and geographic region. The respondents were asked to provide electronic informed consent on the first page of the survey. Korea Research is responsible for protecting the confidentiality of the anonymous survey respondents. Over 1000 participants completed the surveys, of which 990 were included in the analysis after excluding incomplete responses. The participation rate was 62.5%, which is assumed to be acceptable for web-based surveys [27-29]. The present study protocol was reviewed and approved by the Institutional Review Board at Seoul National University (IRB No. 2003/002-005), Seoul, South Korea. All participants, upon enrollment, provided their informed consent. The data collection took place over 3 days (March 25-27, 2020), about 2 months after the Korea Centers for Disease Control and Prevention confirmed the first case of COVID-19 during the early stage of the pandemic and just before 10,000 confirmed cases had been reported (ie, on April 3, 2020).

### Measurements

#### Dependent Variables

Outcome variables were precautionary behaviors related to the threat of COVID-19. They were classified into one of two categories: (1) preventive measures (eg, wearing facial masks and practicing hand hygiene) and (2) social distancing behaviors (eg, avoiding crowded places and postponing or canceling social events). Participants self-reported the frequency of the actions they undertook during the previous week by using a 4-point Likert-type scale (1=never, 2=sometimes, 3=often, and 4=always).

#### Independent Variables

Whether the participants read COVID-19 emergency alert text messages was assessed by the following question: “Do you read the COVID-19 emergency alert text messages received?” Participants responded by choosing one of four options: “I never read the messages” (score=1); “I rarely read the messages” (score=2); “I read the messages occasionally and visit the linked website if necessary” (score=3); or “I always read the message and visit the linked site to get more information” (score=4). Participants who reported they never or rarely read these text messages were also asked why. They were then grouped into binary groups to reduce the likelihood of low content validity due to the survey item; these groups included two behaviors: reading the message and visiting the linked website. The binary groups consisted of (1) participants who always read the text messages and visited the linked website to get more information (score=4) and (2) those who do not (score=1 for “always” and score=0 for “otherwise”).

Among the psychological factors, perceived risk of COVID-19 infection comprised (1) perceived susceptibility, signifying an individual's beliefs about their possibility of infection, and (2) perceived severity, signifying the seriousness of infection [30]. Participants were asked, “What do you think is the possibility of you contracting COVID-19 infection?” and “What do you think will be the severity if you are infected with COVID-19?” Responses were rated on a 5-point Likert-type scale, with responses ranging from “very low” (score=1), “neither low nor high” (score=3), to “very high” (score=5). To promote response efficacy, participants answered the question, “To what extent do you think the precautionary behavior is an effective way to reduce the risk of COVID-19 infection?” for each behavior examined in this study [31]. Their responses were rated on a 4-point Likert-type scale, ranging from “not at all” (score=1) to “extremely” (score=4).

Sociodemographic factors included sex (score=1 for male, score=2 for female), age, and the presence of children younger than elementary school age at home (score=1 for more than 1 child, score=0 for no children). We also assessed the participants’ education level (score=1 for middle school or below, score=2 for high school graduate, score=3 for college and above) and monthly household income reported in their local currency (ie, KRW: score=1 for below KRW 2 million [US $1800], score=2 for KRW 2-3.99 million [US $1800-3600], score=3 for KRW 4-5.99 million [US $3600-5400], and score=4 for KRW 6 million [US $5400] or above). We collected information about the participants’ occupation and whether they were employed, as well as the presence of underlying disease. We also asked the participants to report any diagnoses for the underlying diseases (eg, hypertension, dyslipidemia, diabetes, chronic cardiac disease, asthma, and cancer). We then grouped the participants based on with or without a diagnosis for one or more underlying diseases.

#### Covariates

Other measures associated with the exposure to COVID-19–related alert text messages in other information channels served as covariates in the hierarchical linear regression analysis. Media exposure was measured by the following question: “In the past week, how often did you use media every day to learn about the news?” with answers ranging from “never” (score=1) to “quite often” (score=4). Additionally, we measured the variable of obtaining COVID-19–related information. Participants rated the following questions on a 4-point Likert scale: “Did you actively search for information about COVID-19 during the last week?” with responses ranging from “never” (score=1) to “very often” (score=4).

### Statistical Analysis

All data were weighted by age, sex, and geographic region distributions in South Korea, according to the Korean Statistical Information Service (2019) [32]. The sampling weights were provided by Korea Research, the company that conducted the survey, by using a random iterative method weighting process, in which each participant was assigned a single weight value. Sampling weights were incorporated into all analyses, and corresponding analytic methods were used. Sampling weights were based on all cases from the present study. Statistical analyses were conducted using R version 4.0.2 software (R Foundation for Statistical Computing). All quantitative variables are reported as absolute numbers with proportions (%) and mean values with SD.

The two groups for reading text messages included (1) those who always read and visited the linked website for more information and (2) those who did not. We measured group differences according to participants’ sociodemographic characteristics, psychological factors, and preventive behaviors, which were analyzed by a chi-square test or *t* test, as appropriate (Table 2). A multivariable logistic regression revealed which sociodemographic factors (eg, sex, age, educational level, monthly household income, employment status, presence of young children in the household, and presence of underlying disease) might influence emergency alert text message reading behavior.

**Table 2:** Characteristics of survey participants.

Variables			Participants (N=990)	Weighted^a^ values (N=990)	Participants who read text messages			*P* value^b,c^	
					Always (n=487)	Otherwise (n=503)			
**Sociodemographic factors**									
	**Sex, n** **(%)**								
		Male	475 (47.9)	491 (49.6)	209 (42.8)	282 (56.1)		<.001^b^	
		Female	515 (52)	499 (50.4)	278 (57.2)	221 (43.9)			
	**Age (years), mean (SD)**		47.07 (14.8)	46.45 (15.1)	48.89 (14.0)	44.08 (15.6)		<.001^c^	
		18-29, n (%)	159 (16.1)	176 (17.8)	52 (10.7)	124 (24.7)		<.001^b^	
		30-39, n (%)	157 (15.9)	160 (16.2)	73 (15)	87 (17.3)			
		40-49, n (%)	197 (19.9)	192 (19.4)	103 (21.2)	89 (17.7)			
		50-59, n (%)	205 (20.7)	197 (19.9)	109 (22.4)	88 (17.5)			
		≥60, n (%)	272 (27.5)	264 (26.7)	150 (30.7)	115 (22.9)			
	**Education level, n (%)**								
		Middle school or below	28 (2.8)	29 (2.9)	11 (2.3)	18 (3.6)		.46^b^	
		High school graduate	474 (47.9)	475 (48)	237 (48. 7)	238 (47.3)			
		College and above	488 (49.3)	486 (49.1)	239 (49.1)	247 (49.1)			
	**Monthly household income^d^ (million KRW), n (%)**								
		<2	127 (12.8)	127 (12.9)	67 (13.8)	61 (12.1)		.27^b^	
		2-3.99	312 (31.5)	313 (31.6)	156 (32.1)	157 (31.2)			
		4-5.99	260 (26.3)	259 (26.2)	134 (27.6)	124 (24.7)			
		≥6	291 (29.4)	290 (29.3)	130 (26.5)	161 (32)			
	**Occupation status, n (%)**								
		Out of labor	387 (39.1)	387 (39.1)	287 (59.1)	314 (62.4)		.29^b^	
		Working	603 (60.9)	602 (60.9)	200 (40.9)	189 (37.6)			
	**Presence of children, n (%)** ** **								
		None	894 (90.3)	894 (90.3)	441 (90.6)	453 (90.1)		.79^b^	
		More than 1	96 (9.7)	96 (9.7)	46 (9.5)	50 (9.9)			
	**Underlying disease, n (%)**								
		None	583 (58.9)	590 (59.7)	277 (57)	313 (62.2)		.09^b^	
		More than 1	407 (41.1)	399 (40.4)	210 (43)	190 (37.8)			
**Psychological factors**									
	**Perceived risk, mean (SD)**								
		Perceived susceptibility	2.63 (0.8)	2.62 (0.8)	2.62 (0.8)	2.63 (0.9)		.78^c^	
		Perceived severity	3.68 (0.9)	3.68 (0.9)	3.72 (0.9)	3.63 (0.9)		.16^c^	
	**Response efficacy, mean (SD)**								
		Wearing facial masks	3.69 (0.5)	3.69 (0.5)	3.76 (0.5)	3.62 (0.6)		<.001^c^	
		Hand hygiene	3.78 (0.5)	3.77 (0.5)	3.83 (0.4)	3.72 (0.5)		.002^c^	
		Keeping away from crowded places	3.67 (0.6)	3.66 (0.58)	3.76 (0.5)	3.57 (0.6)		<.001^c^	
		Cancelling or postponing social events	3.70 (0.6)	3.70 (0.6)	3.77 (0.5)	3.62 (0.6)		<.001^c^	
**Practicing precautionary behavior (“always”), n (%)**									
		Wearing facial masks	762 (77.1)	760 (76.8)	400 (82.4)	359 (71. 5)		<.001^b^	
		Hand hygiene	716 (72.3)	714 (72.1)	383 (78.8)	330 (65.6)		<.001^b^	
		Keeping away from crowded places	574 (58)	571 (57.7)	325 (66.9)	246 (48.9)		<.001^b^	
		Cancelling or postponing social events	631 (63.7)	628 (63.4)	354 (72.9)	273 (54.3)		<.001^b^	

^a^Data were weighted by sex, age, and regional distribution of the population in South Korea.

^b^*P* values for chi-square test.

^c^*P* values for *t* test.

^d^A currency exchange conversion rate of KRW 1=US $ 0.00091 is applicable.

Hierarchical linear regressions were computed to estimate the role of reading emergency alert text messages toward each precautionary behavior practiced against COVID-19 by sequentially adding predictors into 3 blocks within each model. To control for the effects of covariates on the dependent variables, sociodemographic factors (ie, sex, age, education level, income level, occupation status, presence of children, and underlying disease), media exposure, and obtaining information were entered into block 1 as potential confounding factors affecting each type of precautionary behavior. Psychological factors such as perceived susceptibility, perceived severity, and response efficacy of each behavior were entered into block 2 of each model. Because we were primarily interested in the effects of reading emergency alert text messages beyond these covariates, predictor variables were subsequently entered into block 3 of each model. To determine whether reading text messages resulted in any significant increment in the amount of variance explained in practicing each precautionary behavior, *F* test statistics were evaluated to determine statistically significant *R^2^* changes in the explained variance (%) at each step of the analysis. Standardized β coefficients were examined for each variable, and variance inflation factor estimates for each hierarchical model were computed to ensure that tolerance estimates were below 0.10 and variance inflation factor estimates were less than 10.

Additionally, the indirect effects of reading the text messages on each precautionary behavior via psychological factors (ie, perceived risk and response efficacy) were calculated using PROCESS macro model 6 with 5000 bootstrap samples for SPSS (version 25; IBM Corp) [33]. The bias-corrected 95% CI values for each mediational path were obtained.

## Results

### Characteristics of Survey Participants

Among the 990 participants, 491 (49.6%) were men and 499 (50.4%) were women, with a mean age of 46.45 (SD 15.05) years. Most participants had received at least some college education (486/990, 49.1%), followed by those with a high school education (475/990, 48%). The most common monthly household income was approximately KRW 2-3.99 million (US $1800-3600), followed by more than KRW 6 million (US $5400) and KRW 4-5.99 million (US $3600-5400). With regard to their occupation status, 60.9% (602/990) of the participants were working, and 39.1% (387/990) were out of labor. Moreover, 9.7% (96/990) of the participants had young children at home, and 40.4% (399/990) of the participants reported having more than one underlying disease (Table 2).

### Psychological Factors and Precautionary Behaviors Related to COVID-19

Participants’ perceived risk of contracting COVID-19 infection was measured by a 5-point Likert scale, and the average perceived susceptibility was higher than “low” (score=2; mean 2.62, SD 0.84). Only 1.7% of the participants reported a perceived chance of infection as “very high” (score=5), and 8.7% of them reported it as “high” (score=4). The majority of participants (490/990, 49.5%) reported that their chance of infection was “neither high nor low.” The average perceived severity score was higher than the perceived susceptibility score, which was close to “high” (score=4; mean 3.68, SD 0.93). However, 42.3% of the participants reported perceived severity as “high” (score=4), and 18.7% of them reported it to be “very high” (score=5). With regard to response efficacy, which was measured on a 4-point Likert scale for the four precautionary behaviors, the response efficacy of practicing hand hygiene was the highest (mean 3.77, SD 0.49), followed by that of canceling or postponing social events (mean 3.70, SD 0.57), wearing facial masks (mean 3.69, SD 0.54), and avoiding crowded places (mean 3.66, SD 0.58), as shown in Table 2.

**Figure 2:**
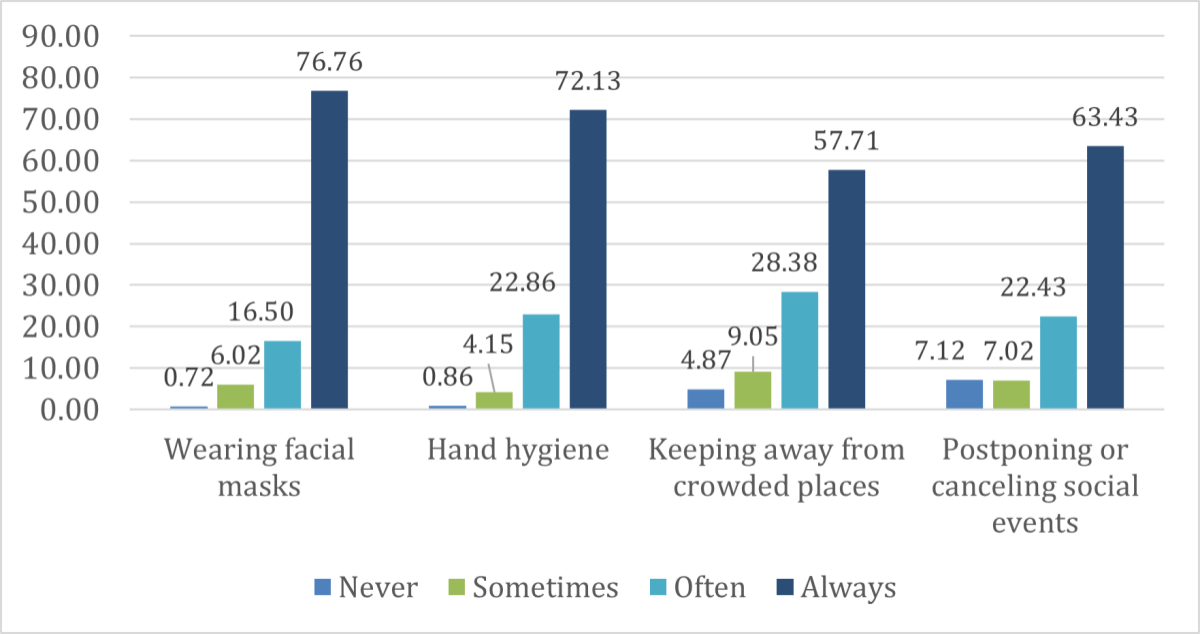
Frequency of practicing precautionary behaviors in the past week (N=990).

The most frequently practiced precautionary behavior was wearing a facial mask when outside, for which 76.8% (760/990) reported they “always” practiced the behavior. For hand hygiene, such as washing hands frequently and using hand sanitizers, 72.1% (714/990) reported they “always” practiced the behavior. With regard to social distancing behaviors, postponing or canceling social events was the most practiced behavior (628/990, 63.4% reporting “always”), followed by avoiding crowded places (571/990, 57.7% reporting “always”), as shown in Figure 2.

### Reading Emergency Alert Text Messages

For reading COVID-19–related emergency alert text messages, 49.2% (487/990) of the participants reported they *always* read the message and visited the linked website to obtain more information. Additionally, 40.3% (399/990) of the participants reported they read the message occasionally and visited the linked website if necessary, 8.4% (83/990) reported they rarely read the message, and 1.9% (21/990) reported they never read the message. Among those who reported they rarely or never read the emergency alert text messages (n=104), most reported not reading the messages because they were “sent too often” (73/104, 70.2%), followed by those who reported “did not want to know the information“ (12/104, 11.5%) or “did not need the information” (9/104, 8.6%).

A multivariable logistic analysis was performed to examine the factors that influence reading emergency alert text messages. Reading text messages was positively associated with female sex (odds ratio [OR] 1.68, 95% CI 1.28-2.21; *P<*.001) and older age (30-39 years: OR 2.02, 95% CI 1.25-3.28; *P<*.001; 40-49 years: OR 2.84, 95% CI 1.80-4.47; *P<*.001; 50-59 years: OR 3.19, 95% CI 2.01-5.06; *P<*.001; ≥60 years: OR 3.12, 95% CI 2.00-4.86; *P<*.001 versus 18-29 years). Men in their 20s comprised the primary sample group that ignored the emergency alert text messages (Table 3). 

**Table 3:** Sociodemographic factors related to reading emergency alert text messages.

Variables		Participants who read text messages (Always=1, otherwise=0)		
		Odds ratio	95% CI	*P* value
**Sex**				
	Male	Ref^a^	Ref	
	Female	1.68	1.28-2.21	<.001
**Age group (years)**				
	18-29	Ref	Ref	
	30-39	2.02	1.25-3.28	<.001
	40-49	2.84	1.80-4.47	<.001
	50-59	3.19	2.01-5.06	<.001
	≥60	3.12	2.00-4.86	<.001
**Education level**				
	Middle school or below	Ref	Ref	
	High school graduate	1.91	0.83-4.36	.13
	College and above	2.10	0.91-4.88	.08
**Monthly household income (million KRW)^b^**				
	<2	Ref	Ref	
	2-3.99	0.89	0.57-1.38	.60
	4-5.99	0.89	0.56-1.41	.61
	≥6	0.67	0.42-1.08	.10
**Occupation status**				
	Out of labor	Ref	Ref	
	Working	0.94	0.69-1.27	.69
**Presence of children**				
	None	Ref	Ref	
	More than 1	0.94	0.59-1.50	.79
**Underlying disease**				
	None	Ref	Ref	
	More than 1	0.95	0.71-1.27	.71
Cox-Snell *R*^2^		N/A^c^	N/A	0.06
Nagelkerke *R*^2^		N/A	N/A	0.08

^a^Ref: reference value.

^b^A currency exchange conversion rate of KRW 1=US $ 0.00091 is applicable.

^c^N/A: not applicable.

### Factors Influencing Practicing Preventive Behaviors

Hierarchical linear regression models were used to test the association of factors influencing practicing preventive behaviors, including perceived risk and response efficacy, and reading emergency alert text messages (Table 4). For wearing facial masks, sociodemographic factors, media exposure, and information seeking (step 1) accounted for 7.4% of the variance and psychological factors (step 2) explained an additional 17.9% of the variance. Adding reading text messages as predictor variable (step 3) explained an additional 0.5% variance for the behavior wearing facial masks (*F*_975_=26.05, *P<*.001). After adjusting for potentially confounding factors, reading text messages were found to be positively associated with wearing facial masks (β=.074, *P=*.012). Overall, the effect of response efficacy of practicing this behavior was found to be significant and the strongest (β=.431, *P*<.001). The positive effect of female sex (β=.108, *P<*.001) and younger age (β=−.064, *P=*.048) were also found to be significant.

**Table 4:** Influencing factors associated with practicing preventive behaviors.

Variables	Wearing facial masks								Hand hygiene							
	Model 1		Model 2			Model 3			Model 1			Model 2			Model 3	
	β^a^	*P* value	β^a^	*P* value	β^a^		*P* value	β^a^		*P* value	β^a^		*P* value	β^a^		*P* value
Sex	.201	<.001	.117	<.001	.108		<.001	.181		<.001	.108		<.001	.101		.001
Age	−.086	.02	−.056	.08	−.064		.048	−.056		.12	−.052		.11	−.057		.08
Education level	.026	.43	.016	.58	.016		.59	.047		.15	.023		.44	.023		.45
Monthly household income	−.004	.90	−.002	.96	.003		.91	.004		.90	.011		.71	.014		.63
Occupation status	−.088	.008	−.053	.08	−.055		.07	−.033		.33	−.007		.81	−.008		.78
Presence of children	−.004	.90	−.005	.86	−.005		.85	−.002		.96	−.012		.68	−.012		.68
Underlying disease	−.037	.28	−.025	.41	−.024		.44	−.034		.33	−.016		.61	−.015		.64
Media exposure	.048	.18	.006	.86	−.004		.89	.133		0	.075		.02	.069		.03
Information seeking	.086	.01	.042	.19	.028		.38	.103		.003	.063		.046	.054		.095
Perceived susceptibility	—^b^	—	.003	.93	.005		.87	—		—	.017		.57	.018		.53
Perceived severity	—	—	.03	.301	.029		.33	—		—	.054		.06	.053		.07
Response efficacy	—	—	.436	<.001	.431		<.001	—		—	.431		<.001	.429		<.001
Reading text messages	—	—	—	—	.074		.01	—		—	—		—	.05		.09
*R* ^2^	0.074	—	0.253	—	0.258		—	0.074		—	0.252		—	0.254		—
Δ*R*^2^	—	—	0.179	<.001	0.005		.01	—		—	0.178		<.001	0.002		0.09
*F* (*df*)	8.677 (979)	<.001	27.543 (976)	<.001	26.05 (975)		<.001	8.692 (979)		<.001	27.465 (976)		<.001	25.618 (975)		<.001

^a^Standardized β coefficients are reported.

^b^Not applicable.

For hand hygiene, sociodemographic factors, media exposure, and information seeking (step 1) accounted for 7.4% of the variance and psychological factors (step 2) explained an additional 17.8% of the variance. Reading text messages (step 3) did not account for significant additional variance in practicing hand hygiene (Δ*R^2^*^=^0.002, *P*=.09; *F*_975_=25.618, *P*<.001). Response efficacy (β=.429, *P<*.001) was found to be significantly associated among the psychological factors. Furthermore, the coefficient of always reading text messages was not significant to practicing hand hygiene.

In terms of the social distancing behavior of keeping away from crowded places, sociodemographic factors, media exposure, and information seeking (step 1) accounted for 5.6% of the variance. Adding psychological factors (step 2) explained an additional 8.9% of the variance. Reading text messages (step 3) explained an additional 0.5% of the variance(*F*_975_=13.253, *P<.*001). Among psychological factors, only response efficacy was found to be associated with the behavior of avoiding crowded places (β=.299, *P<.*001). Reading emergency alert text messages had a significantly positive effect, and participants who reported they always read text messages were more likely to practice avoiding crowded places (β=.078, *P=*.01).

For the social distancing behavior of cancelling or postponing social gatherings, sociodemographic factors, media exposure, and information seeking (step 1) accounted for 3.7% of the variance, and psychological factors (step 2) explained an additional 9.6% of the variance. Adding reading text messages as predictor variable (step 3) explained an additional 0.9% of the variance in wearing facial masks (*F*_975_=12.425, *P<.*001). After adjusting for potentially confounding factors, reading text messages was found to be positively associated with canceling or postponing social gatherings (β=.103, *P=.*001). The effect of response efficacy of practicing this behavior was significant and the strongest (β=.306, *P<*.001), as shown in Table 5.

**Table 5:** Influencing factors associated with practicing social distancing behaviors.

Variables	Keeping away from crowded places						Canceling or postponing social events						
	Model 1		Model 2		Model 3		Model 1		Model 2		Model 3		
	β^a^	*P* value	β^a^	*P* value	β^a^	*P* value	β^a^	*P* value	β^a^	*P* value		β^a^	*P* value
Sex	.079	.02	.03	.35	.021	.52	.064	.06	.024	.46		.011	.74
Age (year)	.005	.89	.004	.91	−.004	.91	.018	.63	.023	.51		.013	.71
Education level	.029	.38	.039	.22	.038	.23	.03	.38	.043	.18		.042	.19
Monthly household income	.03	.37	.023	.47	.028	.37	.03	.37	.012	.70		.02	.54
Occupation status	−.052	.12	−.052	.11	−.053	.095	−.051	.13	−.038	.24		−.041	.21
Presence of children	.018	.57	.007	.82	.007	.82	−.005	.87	−.025	.41		−.025	.41
Underlying disease	.005	.88	.022	.51	.023	.48	.008	.82	.022	.52		.024	.48
Media exposure	.087	.02	.057	.09	.047	.17	.074	.04	.035	.30		.022	.53
Information seeking	.147	<.001	.102	.003	.088	.01	.105	.003	.06	.08		.041	.23
Perceived susceptibility	—^b^	—	.007	.82	.009	.77	—	—	.026	.40		.029	.35
Perceived severity	—	—	.026	.39	.025	.42	—	—	.055	.08		.053	.09
Response efficacy	—	—	.306	<.001	.299	<.001	—	—	.313	<.001		.306	<.001
Reading text messages	—	—	—	—	.078	.01	—	—	—	—		.103	.001
*R* ^2^	.056	—	.145	—	.15	—	.037	—	.133	—		.142	—
Δ*R*^2^	—	—	.089	<.001	.005	.01	—	—	.096	<.001		.009	.001
*F* (*df*)	6.462 (979)	<.001	13.779 (976)	<.001	13.253 (975)	<.001	4.152 (979)	<.001	12.47 (976)	<.001		12.425 (975)	<.001

^a^Standardized β coefficients are reported.

^b^Not applicable.

### Mediation Effect of Psychological Factors Between Reading Text Messages and Practicing Preventive Behaviors

We examined the direct and indirect effects of reading text messages that were mediated via psychological factors such as perceived risk and response efficacy of practicing behaviors. When it comes to the recommended behavior, the direct and indirect effects of reading text messages on wearing masks was significant. For hand hygiene, only the indirect effect, mediated via response efficacy of the behaviors, was significant. Concerning social distancing, the direct and indirect effects of reading text messages mediated by the response efficacy of the behavior were also significant (Table 6). Therefore, this result confirms the significant effect of reading text messages on practicing precautionary behavior.

**Table 6:** Direct and indirect effects of reading emergency alert text messages on practicing preventive behaviors based on perceptions (eg, perceived susceptibility, severity, and response efficacy). Unstandardized point estimates represent the indirect effect of the independent variable on the dependent variable through the mediator.

Dependent variable	Preventive behaviors			Social distancing behaviors	
	Wearing facial masks	Hand hygiene	Keeping away from crowded places		Canceling or postponing social events
	Estimate (95% CI^a^)	Estimate (95% CI)	Estimate (95% CI)		Estimate (95% CI)
Total effect	0.1257 (0.0475 to 0.2039)	0.0795 (0.0031 to 0.1558)	0.1772 (0.0682 to 0.2861)		0.2190 (0.1020 to 0.3361)
Direct effect	0.0887 (0.0178 to 0.1595)	0.0602 (−0.0088 to 0.1292)	0.1276 (0.0228 to 0.2323)		0.1804 (0.0684 to 0.2924)
Indirect effect (via perceived susceptibility)	−0.0001 (−0.0039 to 0.0035)	−0.0006 (−0.0047 to 0.0027)	−0.0004 (−0.0061 to 0.0043)		−0.0014 (−0.0084 to 0.0037)
Indirect effect (via perceived severity	0.0005 (−0.0029 to 0.0047)	0.0009 (−0.0036 to 0.0063)	0.0006 (−0.0039 to 0.0063)		0.0012 (−0.0046 to 0.0095)
Indirect effect (via response efficacy)	0.0366 (0.0041 to 0.0705)	0.019 (0.0134 to 0.0508)	0.0494 (0.0161 to 0.0847)		0.0389 (0.0043 to 0.0769)

^a^Bias-corrected CI (these 95% CIs do not cross zero; thus, mediation is assumed).

## Discussion

### Principal Findings

In this study, the effects of reading emergency alert text messages on practicing preventive behaviors during the COVID-19 outbreak in South Korea were examined. Reading text messages was found to be related to all precautionary behaviors tested, including wearing facial masks, practicing hand hygiene, avoiding crowded places, and avoiding social gatherings. Reading text messages directly or indirectly affected practicing precautionary behaviors, as the mediation effect of response efficacy between reading text messages and practicing those behaviors was significant. Sociodemographic factors (eg, male participants in their 20s) were related to a lower likelihood of reading emergency alert text messages.

Several findings provide valuable insights to the public health management authorities to prevent and protect the population's health during an emerging infectious disease outbreak. First, our study results indicate that health risk communication via emergency alert text messages is efficient and effective for engaging the public in practicing preventive behaviors during public health emergencies. Participants who always read and visited the linked website practiced precautionary behaviors 1.48-1.80 times more frequently than other participants. This effect was significant, even when media use was included in the logistic model as a covariate. Early release of official guidelines and timely provision of information led by governments to guide the public on responding to emergencies is essential during public health emergencies [34,35]. Therefore, mobile phones, especially text messages, can be an effective means of communication to elicit the rapid response required by public health authorities.

The phenomenon of “infodemics,” defined as the rapid spread and amplification of vast amounts of valid and invalid information on the internet or through other media, is a tremendous and ongoing challenge on the COVID-19 pandemic [36,37]. Since the beginning of the COVID-19 pandemic, both the production and consumption of information have increased rapidly and significantly [9,12]. According to a study conducted in Korea, more than two-thirds of the participants reported exposure to COVID-19 misinformation between January and April 2020 [38]. Communication via emergency alert text messages can be an effective strategy for public health authorities to provide accurate and reliable information, confront misinformation or disinformation, and reduce the negative impact of such infodemics. Additionally, digital inequality, also known as the digital divide, is a significant concern among national and international scholars and policy makers, as internet access such as material, skills, and usage is not evenly distributed among the general population [39,40]. People can receive text messages on their mobile phones without using a smartphone or connecting to the internet. Therefore, text messages can be a communication tool to alleviate digital inequalities.

Second, some people rarely or never read text messages. The present study identified a subpopulation that does not pay attention to the text messages sent by the public health authorities. People often ignore the emergency warning and alert messages as initial responses are often marked by skepticism, disbelief, and denial [41-43]. Explanations for the lack of response include repeated, long-term exposure to a warning that may contribute to message fatigue and a loss of ability to capture and maintain the attention needed to elicit a response at later times [25,44-46]. Kuligowski and Dootson [35] proposed a 6-step process to process information and respond. These steps include (1) receiving the information, (2) paying attention to the message, (3) aiming to understand the information provided, (4) believing the threat presented by the message, (5) personalizing the presented risk to themselves or others, and finally, (6) deciding to take protective action and to respond based on the information received in the message [19,35,47]. Their study indicates that a high level of attention to the provided information is essential for the recipient to move to the next step. Hence, convincing the public that reading emergency alert text messages can help guide them to better respond to public health emergencies is important.

Considering the COVID-19 emergency has lasted about a year and seems likely to continue for some time, more people will likely not pay attention to text messages in the future. Therefore, further research is needed to identify the reasons for not paying attention to text messages and investigate how to maintain interest. The content, length, frequency, and timing of the alert should be reviewed to maximize the effectiveness of emergency alert text messages. Moreover, people with limited literacy might resist to read or be attentive toward these text messages. Using a symbol and number-coded system that is easy to remember and interpret by the public, even among those with limited literacy, can be considered to address this challenge [48]. As communication can help reduce health inequalities derived from health communication [49], efforts to reduce vulnerability should also be implemented.

This study also identified a subpopulation that read alert text messages less frequently than others. Men in their 20s were the least likely to read the text messages they received. Infrequent reading of text messages by participants in their 20s might be because they are more proficient in using mobile technology and prefer obtaining information via online digital resources. However, their digital health literacy—the ability to evaluate health resources and apply gathered information to health-related decisions—was relatively low [38,50]. Therefore, young people have less opportunity to obtain official information from public health authorities and are more likely to be exposed to misinformation distributed online. This disparity can make them more vulnerable to the COVID-19 infodemic. Additionally, current practice in emergency alert text messages sends the same message, regardless of the recipient's demographic characteristics. To enhance the efficacy of text messages, tailored messages for each population and subgroups can be considered.

Finally, reading text messages in this study was mediated through heightened response efficacy of practicing behaviors rather than perceived risk. Moreover, response efficacy is one factor that has the strongest positive effect on practicing preventive behaviors [13,51,52]. Public health authorities and policymakers can consider making efforts to improve messages sent via texting to strengthen response efficacy on practicing preventive behaviors. For instance, investigating the unmet needs of health risk information and providing for them can be helpful. In other words, receiver-centered messages should be provided. We propose that future studies analyze the messages sent by texting to design more effective messages.

### Implications

A set of implications for interventions, communication strategies, and future research can be drawn from the findings of this study. Governments, health agencies, and researchers should take advantage of mobile text messages by producing and sharing evidence-based and correct information, as well as recommending precautionary behaviors to the public. Public health authorities should pay careful attention to what messages are provided to the public. Based on the findings of this study, we believe that persuasive messages that target to improve response efficacy on recommended behaviors would be most useful.

Second, for effective text message communication, the public needs a moderate or higher trust level in the government and health authorities [48]. Successful risk communication and efficacy of policy recommendations depend not only on how quickly or reliably a message can be disseminated but also on the individuals' beliefs and subsequent response [6,15,16,53,54]. The first step in this process is the firm belief of whether the recommended action will mitigate the threat or manage the fearful situation [54].

Third, health authorities' efforts to provide transparent and credible information should be encouraged and sustained. South Korea has prior experience in managing outbreaks of infectious diseases such as the MERS outbreak in 2015, which resulted in significant damage to the Korean population, widespread distrust, and societal levels of high stress. Nevertheless, the MERS pandemic has provided many important lessons, especially the importance of information disclosure to the public [13]. In case of the MERS outbreak, the Korean health authorities' decision to disclose specific information, such as hospitals that were exposed to MERS-CoV, contributed to further prevention of the spread of infection during the outbreak [55].

### Limitations

The present study has several limitations. First, variables in this study were assessed using a single-item questionnaire. Single items are less time-consuming, minimize participant burden, and prove beneficial to surveys that need to be done quickly to provide timely information. Single-item measures are argued to have comparable or equal predictive validity compared to multiple-item measures in some study areas [56]. However, this approach can be problematic because of the unknown biases in meaning and interpretation, and the internal consistency, as well as reliability statistics, cannot be tested. Therefore, additional studies using multi-item questionnaires should be performed.

Second, the participants did not all receive the same text messages. As shown in Table 1, the central governments' messages were the same for all of the population, and the contents were mostly about public health policy updates, persuading practice of precautionary behaviors. The messages sent from local governments usually covered more residence-specific risk information, such as reports of confirmed cases in the area, contact tracing of confirmed cases, and closure of certain places. This study was not designed to test the effect of specific messages, like previous experimental studies [57-59]. There is still a chance that differences in how people are primed might influence their preventive behaviors. Nevertheless, this study has the advantage of being conducted on many people in real-life situations. This study's results should be interpreted focusing on the effect of using emergency alert text messages as one of the information sources, rather than focusing on the effect of the content of the messages received. 

Third, we did not explore self-efficacy in this study. Self-efficacy refers to the individual's level of confidence in preventing the risk and is an essential coping appraisal component [54,60]. Thus, further study to examine the influence of self-efficacy is suggested. Lastly, the participants ' characteristics, such as health literacy, government trust, or perceived credibility on the text messages, were not explored in this study. These variables may contribute to both whether and how messages were read and whether precautionary behaviors were practiced. The effects of these variables should also be examined in further study.

### Conclusions

New information technologies, communication devices, apps, and social media for health risk communication are continually emerging. However, sometimes *oldies are goodies*, which this study implicates. The present study provides evidence that emergency alert text messages sent to an individual's mobile phone are efficient and an effective communication strategy for the sustainability of preventive behaviors among the public. Government and public health authorities should use text messages to provide the population with accurate and reliable information. This approach can reduce the negative impact of an infodemic by confronting misinformation or disinformation. At the same time, efforts to ensure people keep reading text messages should be implemented. This study provides critical and timely insights into how governments and public health authorities build appropriate health risk communications that do not overlook and lower their priorities for those in urgent need.

## Abbreviations

KCDC: Korea Centers for Disease Control and Prevention
MERS: Middle East Respiratory Syndrome
OR: odds ratio
