# Correction: Effects of COVID-19 Emergency Alert Text Messages on Practicing Preventive Behaviors: Cross-sectional Web-Based Survey in South Korea

**DOI:** 10.2196/28660

**Published:** 2021-03-18

**Authors:** Minjung Lee, Myoungsoon You

**Affiliations:** 1 Department of Public Health Sciences, Graduate School of Public Health Seoul National University Seoul Republic of Korea; 2 Office of Dental Education, School of Dentistry Seoul National University Seoul Republic of Korea

In “Effects of COVID-19 Emergency Alert Text Messages on Practicing Preventive Behaviors: Cross-sectional Web-Based Survey in South Korea” (J Med Internet Res 2021;23(2):e24165) the authors noted an error in authorship.

In the originally published article, the order of authors was incorrectly listed as follows:

Myoungsoon You, Minjung Lee

The authorship list has been corrected to:

Minjung Lee, Myoungsoon You

The correction will appear in the online version of the paper on the JMIR Publications website on March 18, 2021, together with the publication of this correction notice. Because this was made after submission to PubMed, PubMed Central, and other full-text repositories, the corrected article has also been resubmitted to those repositories.

